# Conscious Presence and Self Control as a measure of situational awareness in soldiers – A validation study

**DOI:** 10.1186/1752-4458-7-1

**Published:** 2013-01-07

**Authors:** Arndt Büssing, Harald Walach, Niko Kohls, Fred Zimmermann, Marion Trousselard

**Affiliations:** 1 Quality of Life, Spirituality and Coping, Center for Integrative Medicine, Faculty of Health, Witten/Herdecke University, Herdecke, Germany; 2 Institute for Transcultural Health Studies and European Office of the Samueli Institute, Europa University Viadrina, Berlin, Germany; 3 Generation Research Program, Human Science Center, University of Munich, Bad Tölz, Germany; and Brain, Mind & Healing Program, Samueli Institute, Alexandria, USA; 4 Neurophysiology of Emotions, Département des Facteurs Humains, Centre de recherches du service de santé des Armées, La Tronche Cédex, France

**Keywords:** Mindfulness, Conscious presence, Soldiers, Trauma, Validation, Questionnaire

## Abstract

**Background:**

The concept of `mindfulness´ was operationalized primarily for patients with chronic stressors, while it is rarely used in reference to soldiers. We intended to validate a modified instrument on the basis of the Freiburg Mindfulness Inventory (FMI) to measure soldiers’ situational awareness (“mindfulness”) in stressful situations/missions. The instrument we will explore in this paper is termed the *Conscious Presence and Self Control* (CPSC) scale.

**Methods:**

The CPSC and further instruments, i.e., Perceived Stress Scale (PSS), stressful military experiences (PCL-M), life satisfaction (BMLSS), Positive Life Construction (ePLC), and self-perceived health affections (VAS), were administered to 281 German soldiers. The soldiers were mainly exposed to explosive ordnance, military police, medical service, and patients with posttraumatic stress disorders.

**Results:**

The 10-item CPSC scale exhibited a one-factorial structure and showed a good internal consistence (Cronbach´s alpha = .86); there were neither ceiling nor bottom effects. The CPSC scores correlated moderately with Positive Life Construction and life satisfaction, and negatively with perceived stress and health affections. Regression analyses indicated that posttraumatic stress disorder symptoms (negative), and the development of effective strategies to deal with disturbing pictures and experiences (positive) were the best predictor of soldiers´ CPSC scores. Soldiers with health affections exhibiting impact upon their daily life had significantly lower CPSC scores than those without impairment (F=8.1; p < .0001).

**Conclusions:**

As core conceptualizations of `mindfulness´ are not necessarily discussed in a military context, the FMI was adopted for military personnel populations, while its two factorial structure with the sub-constructs `acceptance´ and `presence´ was retained. The resulting 10-item CPSC scale had good internal consistence, sound associations with measures of health affections and life satisfaction, and thus can be used as a short and rapid measure in pre-post mission and interventional studies.

## Background

The concept of `mindfulness´, which has now become an integral part of mind-body medicine, was operationalized primarily for patients suffering from chronic pain and stress, and is until now rarely used in military contexts. However, research has suggested that a similar health component, “mind fitness training” in soldiers, operationalized as “mental agility, emotional regulation, attention and situational awareness” [1] may be protective in building resilience and leading to faster recovery from psychological stress. Mind fitness comprises various training components, including mindfulness based interventions such as the Mindfulness-based Stress-Reduction [MBSR] programs. Bates *et al.*[2] argued that spiritual and psychological fitness are inter-related, and highlighted that mindfulness, as a specific form of spiritual practices and mind-body training, may improve attention and self-regulation.

Several meta-analyses indicate that mindfulness based interventions and programs may both improve mental stability and coping with stress [3-6]. In cancer patients, MBSR programs improve patients´ mental health with moderate effect sizes, while the impact on physical health effects is rather weak [4]. A meta-analysis of Musial *et al.*[6] additionally found that MBSR programs can improve quality of life and mood, and reduce distress in cancer patients. However, the reported effects were found to be strong only for stress reduction, moderate concerning emotional regulation and rather impacting upon generic quality of life [6]. In healthy individuals, Chiesa and Serretti [5] observed that mindfulness based interventions may reduce stress, ruminative thinking and trait anxiety as well as increase empathy and self-compassion. To attenuate stress and concerns - factors known to hamper overall sleep quality -, MBSR programs have been used as interventions to improve self-management and to reduce or reframe intrusive thoughts. The systematic review of Winbush *et al.*[3] found “some evidence” that mindfulness techniques may attenuate sleep-interfering cognitive processes and could thus improve sleep disturbances. Praissman´s 2008 literature review and clinician´s guide [7] stated that “MBSR is a safe, effective, integrative approach for reducing stress”, with beneficial effect to enhance the interaction between healthcare providers and patients.

However, these findings were predominantly observed in clinical and nonclinical civilian populations, and it is currently unclear whether I) mindfulness programs are accepted by (predominantly male) soldiers and military personnel, II) are of relevance to improve resilience, performance and coping in/with critical (combat theatre and peacekeeping mission) situations. Some studies indicate that MBSR may improve posttraumatic stress disorder (PTSD) symptoms such as depression, behavioural activation, and acceptance [8,9]. In a randomized controlled study enrolling veterans, Kearney et al. [8,9] found a significant six month effect for a MBSR program on PTSD symptoms, depression, behavioral activation, mental quality of life, acceptance and mindfulness [8] with medium to large effect sizes for depression, mental health-related quality of life [HRQOL], and mindfulness skills” [9]. A similar study with fire fighters also implied that mindfulness may be associated with fewer posttraumatic stress disorder [PTSD] symptoms, depressive symptoms, physical symptoms, and alcohol problems [10]. A concept very similar to MBSR called “Mindfulness-based Mind Fitness Training” [MMFT], was tested in a pilot study in 31 US Marine Reservists before they were deployed to their combat theatre [1]. The authors stated that “some Marines resisted the effort required by the training”, while others have “personalized their approach to the MMFT exercises”. Stanley and Jha [1] reported that those who spent more time in their mind fitness exercises showed an improvement in their cognitive performance, while those soldiers who spent less time engaging in these practices showed an increase of their perceived pre-deployment distress levels. Interestingly, more than half of population studied reported to have used the MMFT skills during their period of deployment.

Although the concept of `mindfulness´ originates in ancient Buddhist philosophy, MBSR and other interventions have been developed and operationalized as secular interventions in the context of the modern Western medical system and secular societies [11-13].

Mindfulness refers to the ability to experience the given situation in a non-judgmental way, in a state of consciousness that allows an individual to be fully present and aware of arising emotions and feelings, conflicting memories and disturbing worries, which could distract the conscious awareness and concentration. Although mindfulness focuses on the present situation, it is not meant as a technique to actively control the situation - instead it allows one to be present with full awareness and attention (whatever the underlying emotional components might be) [14]. Bishop *et al.*[15] stated that they regard “mindfulness as a process of regulating attention in order to bring a quality of non-elaborative awareness to current experience and a quality of relating to one’s experience within an orientation of curiosity, experiential openness, and acceptance”. Mindfulness training therefore also allows an individual to deal with distracting or ambivalent emotions and feelings, conflicting memories and disturbing worries that may arise in stressful situations. Empirical research has identified two interrelated sub-facets of mindfulness, i.e., `awareness/presence´ and `openness/acceptance´ [16,17]. While `presence´ refers to the cognitive capacity to focus on the experience of the present moment, `acceptance´ pertains to the emotional ability to control the automatically generated emotional responses to a given situation so that an individual’s attention is not dragged away by uncontrolled emotions.

Although mindfulness is not a term commonly used in a military context, it is of interest to note that the term “situational awareness”, which is a pivotal construct in military psychology and information warfare, shares some common conceptual ground with mindfulness. However, in contrast to mindfulness, which comprises both the milieu *intérieur* and *extérieur,* situational awareness only refers to the sensual awareness of the outer world. Situational awareness has been defined as “the perception of the elements in the environment within a volume of space and time, the comprehension of their meaning, the projection of their status into the near future, and the prediction of how various actions will affect die fulfillment of one´s goals” by Endsley [18]. The ability to acquire situational awareness skills may be moderated by individual mindfulness levels. Recently the concept of “mind fitness” was introduced, suggesting that the ability to be present and to self-regulate emotions may be helpful to enhance soldiers´ competencies to react adequately in critical situations, to manage better with stress, and to improve resilience and recovery from psycho-emotional affections and impairment [19]. A similar concept (“Warrior Spirit”) was brought up by Strozzi-Heckler [17] who investigated the impact of awareness training in 25 Green Berets, and reported an improvement of their skills to view “themselves in relation to the world around”.

To measure mindfulness, several instruments have been published [20], but not all of them have been validated extensively [21]. Among the relevant instruments are the *Mindfulness Attention and Awareness Scale*[22] and the *Freiburg Mindfulness Inventory* (FMI; [23]). Particularly the shortened 14-item version of the FMI is widely used and seems to be a promising instrument. The primary 30-item version of the FMI was tested among participants of a mindfulness meditation retreat and had a very good internal consistency (Cronbach´s alpha = .93); also the 14-item version of the FMI, which was tested in a sample of individuals with and without meditation experience, and individuals with clinical problems, had a good psychometric quality (alpha = .86) [23]. For this instrument, the authors suggested a one-factorial [23] and a two factorial solution [24]. Recent Rasch analysis of data obtained from patients with different psychosomatic conditions as well as a large non-clinical population indicated the possibility of extant bottom and ceiling effects [25]. Additionally, Rasch analysis suggested that a 13-item version with two subfactors would be more appropriate, while the one-factor solution seems to be less appropriate [25-27]. These two factors can be described as the `presence´ and `acceptance´ subfacet of mindfulness. Rasch analysis indicated that the item difficulty of the items referring to the `presence´ component was lower than that of the `acceptance´ items [25]. The authors therefore suggested that “the ability to be present is established before the ability to accept things” can be learnt.

However, several items of the FMI are difficult to understand, particularly for those individuals who have no experience with mindfulness meditation and the underlying concepts. Belzer et al. [28] have shown that participants with lack of mindfulness experience had greater difficulties with item comprehension than individuals with mindfulness experience. The authors therefore recommended “a modification of the wording of several FMI items”, at least for populations with lack of familiarity with the concept of mindfulness as they opine there may be “insufficient construct validity to use the current FMI in mindfulness-naïve samples” [28].

Indeed, as preliminary studies with the FMI have clearly revealed that military personnel and soldiers have difficulties with understanding at least some items of the FMI-14, we aimed to develop and validate a modified instrument on the basis of the FMI that is specifically tailored to military personnel. This is achieved by shifting the conceptual focus to the conscious presence and perception of a given situation as well as the attentional self-control of individuals in stressful situation. In this paper, we describe the construction and validation of the *Conscious Presence and Self Control* (CPSC) scale as a measure of situational awareness in soldiers with and without psycho-emotional affections. This implies the analysis of variables with an influence on CPSC scores, i.e. socio-demographic variables and health affections.

## Methods

### Participants

All individuals of this anonymously conducted cross-sectional study were informed about the purpose of the study, were assured of confidentiality and their right to withdraw at any time, and asked to provide informed consent. Ethical approval was obtained by the IRB of Witten/Herdecke University (#109/2011), and relevant officials of the German Ministry of Defense endorsed the realization of the study.

The questionnaires were administered to German soldiers (mainly explosive ordnance disposal unit, military police/personal security, medical services) and military personnel treated as in-patients for posttraumatic stress disorders in the German Armed Forces Military Hospital Hamburg (Department of Psychiatry and Psychotherapy). With the exception of the clinical sample patients, for this study we exclusively focused on soldiers with `helping and assisting´ duties, because these troops were trained to provide care and shelter for other units. Apart from this, we had no strict inclusion or exclusion criteria and all soldiers were asked to provide anonymized data on a voluntary basis.

With the aforementioned criterion in place, 281 individuals provided data leading to a response rate of 38%.

Socio-demographic data of the study participants is given in Table 1.

**Table 1:** Characteristics of 281 individuals

**Variables**	Number (%)*/means (range)
**Gender,** number (%)	
Men	266 (95)
Women	13 (5)
**Age, years** (Mean, standard deviation)	31.9 ± 8.7
**Family status**, number (%)	
Married	111 (40)
Living with partner	82 (30)
Single	65 (24)
Widowed	15 (6)
**Educational level**, number (%)	
secondary (Hauptschule)	41 (15)
junior high school (Realschule)	163 (59)
high school (Gymnasium)	59 (21)
other	13 (5)
**Religious orientation,** number (%)	
Christian	166 (60)
Other	4 (1)
None	108 (39)
**Health associated variables** (mean ± SD, range)	
Perceived Stress (PSS-6)	15.2 ± 5.8 (6–30)
PTSD scores (PCL-M)	29.6 ± 14.4 (0–79)
Self perceived health affection (NRC)	19.9 ± 26.1 (0–100)
Life Satisfaction (BMLSS-10)	70.1 ± 15.6 (12–100)
Positive Life Construction (ePLC)	69.8 ± 22.3 (0–100)

* with respect to gender (n=2), family status (n=8), educational level (n=5), and religious denomination (n=3), some did not provide the respective information. In these cases, the relative proportions were referred to the number of respondents.

### Measures

The questionnaire battery consisting of six scales was administered between November 2011 and January 2012. Data entry was performed at the German Armed Forces Joint Support Command, Cologne, while the anonymized data set was analyzed at the Witten/Herdecke University. The following psychometric instruments were used in their respective German versions:

#### Conscious presence and self control (CPSC) scale

We intended to design an instrument with the ability to measure military personnel’s conscious presence and perception of a given situation and their self control in difficult situations. As the underlying construct can be described as mindfulness and situational awareness in military personnel, we referred to the 14-item version of the FMI [23]. The authors stated that the short 14 item scale “is sensitive to change and can be used also with subjects without previous meditation experience” [23]. Nevertheless, several specific item phrasings of the FMI are difficult to understand or are less appropriate for individuals with lack of mindfulness training and resulting unfamiliarity of the underlying concepts. Therefore, we specified and adjusted the pivotal items of the FMI to the military context using specific explanations, or alternatively reformulated items where appropriate. Additionally, four items (i.e. items 4, 5, 13 and 14) had to be removed as they were considered to be inappropriate for a military context. As can be seem in Table 2, eight items were reformulated, eight were removed and two remained unchanged. Response options were `rarely´ (0), `occasionally´ (1), `fairly often´ (2), and `almost always´ (3). Data are given as mean scores. The resulting item pool was then submitted to empirical investigation as the *Conscious presence and self control* (CPSC) scale, and tested for its psychometric properties.

**Table 2:** Comparison of original items and modified/new items of the CPSC scale

	**Original FMI items**	**Modified/new CPSC items**
1	I am open to the experience of the present moment. (P)	I consciously perceive my current situation and can look at it (as if from the outside) without judging it as either 'good' or 'bad'.
2	I sense my body, whether eating, cooking, cleaning or talking. (P)	With everything I do (eating, cleaning, conversations, official duties, etc.) I am always conscious of the emotions, moods and physical responses that occur.
3	When I notice an absence of mind, I gently return to the experience of the here and now. (P)	Once I realize that my concentration has drifted I can return to consciously paying attention without difficulties.
4	I am able to appreciate myself. (A)	[deleted]
5	I pay attention to what’s behind my actions. (P)	[deleted]
6	I see my mistakes and difficulties without judging them. (A)	I see my mistakes and difficulties without judging them.
7	I feel connected to my experience in the here-and-now. (P)	In everything I do I am paying full attention and perceive everything mindfully.
8	I accept unpleasant experiences. (A)	I also accept unpleasant experiences as being important and of value.
9	I am friendly to myself when things go wrong. (A)	I am friendly to myself when things go wrong.
10	I watch my feelings without getting lost in them. (A)	In difficult situations I can observe any kind of arising emotions from a distance without getting lost in them.
11	In difficult situations, I can pause without immediately reacting. (A)	In difficult situations I do not let arising emotions take control over me.
12	I experience moments of inner peace and ease, even when things get hectic and stressful. (A)	I experience moments of inner peace and serenity even if faced with pain and disturbances or when I am dealing with difficult situations.
13	I am impatient with myself and with others. (A)	[deleted]
14	I am able to smile when I notice how I sometimes make life difficult. (A)	[deleted]

P - refers to the underlying 'presence' component; A - refers to the underlying 'acceptance' component.

#### Life Satisfaction

Life satisfaction was measured using the *Brief Multidimensional Life Satisfaction Scale* (BMLSS) [29]. The items of the BMLSS address intrinsic (oneself, life in general), social (friendships, family life), external (work situation, where one live) and prospective dimensions (financial situation, future prospects) of life satisfaction as a multifaceted construct. The internal consistency of the instrument was found to be good in the validation study (Cronbach’s alpha = .87) [29]. In this study, the 10-item version was employed that includes satisfaction with the health situation and abilities to deal with daily life concerns (BMLSS-10). The scale exhibited a good internal consistency in the given population (alpha= .83).

The BMLSS was complemented by five additional items, which addressed satisfaction with the team, i.e. support by team comrades and team leader, appreciation by team comrades and team leader, and team spirit. These five items collapse into a single *Satisfaction with Team* scale that showed a good internal consistency (alpha = .89).

Each of these 15 items was introduced by the phrase ‘I would describe my level of satisfaction as …’, and scored on a 7-point scale ranging from dissatisfaction to satisfaction (0 – terrible; 1 – unhappy; 2 – mostly dissatisfied; 3 – mixed (about equally satisfied and dissatisfied); 4 – mostly satisfied; 5 – pleased; 6 – delighted). The BMLSS-10 sum scores refer to a 100% level (`delighted´). Scores > 50% indicate higher life satisfaction, while scores < 50% indicate dissatisfaction.

#### Positive life construction

The *Positive Life Construction* (ePLC) scale was taken from the Emotional and Rational Disease Acceptance (ERDA) questionnaire and can be interpreted as an indicator of an emotional acceptance strategy [30,31]. Previous research has shown that this scale has a good to very good internal consistency (Cronbach’s alpha between .86 and .92) [30,31]. Moreover substantial correlations with external health criteria, i.e., depression, anxiety, hostility, obsessive-compulsive orientation, somatization, and life satisfaction were found [31]. Within this population of soldiers, Cronbach´s alpha of the scale was .87.

The eight items were scored on a five point Likert scale ranging from `does not apply at all´ (0) to 'applies very much´ (4). The respective sum score was transformed to a 100% level (transformed scale score).

#### Perceived stress scale

The Perceived Stress Scale (PSS) measures a person´s self-perceived stress level in specific situations during the last month [32]. Four items of the 10-item version (PSS-10) use a reverse scoring. Internal reliability of the original PSS-10 was moderate (alpha = .78) [32]. Here we used the German version of the scale, which consist of 6 items (due to a week item to scale correlation, 3 negative items had to be excluded from the item pool, i.e. items 4, 5, 7, while a further negative item was excluded too). Within this sample, the German language PSS-6 has a good internal reliability (alpha = .89).

All items refer to emotions and thoughts and how often one may have felt or thought a certain way. The scores range from 1 (never) to 4 (very often); higher scores would thus indicate greater stress.

#### Stressful military experiences/post-traumatic stress disorders

Stressful military experiences in terms of post-traumatic stress disorders (PTSD) were measured with the German version of the PTSD Checklist-Military Version (PCL-M) [33]. The checklist addresses problems associated with psychological distress that soldiers and veterans may experience, i.e., repeated, disturbing memories, thoughts, images or dreams of a stressful military experience, physical reactions when something reminded of a stressful military experience, avoidance of activities or situations because they reminded of a stressful military experience, being “superalert” or watchful or on guard, etc. [34,35].

The internal validity of the 17-item German PCL-M was established as very high (alpha = .93); only one item (#5) had a weak item–scale correlation (.37) and could have been removed from the item pool. Further details on the validation of the German version of the instrument are in preparation.

The respective items were scored on a 5-point Likert scale ranging from 1 (not at all) to 5 (extremely). The total `symptom severity score´ may range from 17 to 85. We did not use the checklist to diagnose PTSD, but to screen individuals for perceived stressful experiences.

#### Self perceived health affection

Soldiers´ self perceived health affections were measured with a visual analogue scale (VAS). We asked for health impairing affections and symptoms within the last weeks, ranging from 0 (none) to 100 (unbearable).

### Statistical analysis

Descriptive statistics, internal consistency (Cronbach’s coefficient α) and factor analyses (principal component analysis using Varimax rotation with Kaiser’s normalization), as well as analyses of variance, first order correlations and regression analyses were computed with SPSS 20.0. Given the exploratory character of this study, significance level was set at p < .05. With respect to classifying the strength of the observed correlations, we regarded r > .5 as a strong correlation, an r between .3 and .5 as a moderate correlation, an r between .2 and .3 as a weak correlation, and r < .2 as no or a negligible correlation.

## Results

### Participants

Most participants were male and were living with a partner; their mean age was 32 ± 9 years (Table 1). Among them, 15% were treated as patients in the Department of Psychiatry and Psychotherapy of German Armed Forces Military Hospital Hamburg (PTBS and other comorbidities). The non-clinical sample can be categorized as explosive ordnance (32%), military police / personal security (23%), medical service (5%), others (40%).

Deployment experience was heterogeneous: 25% had currently participated in only one mission, 22% had two, 11% had three, 11% had four, 5% had five, and 9% participated between six and fourteen missions; 17% did not answer the question.

Perceived Stress scores of the military personnel enrolled in this study were in a medium range, while self perceived health affections and also PTSD scores were in the lower range; their life satisfaction and *Positive Life Construction* scores were in the upper range (Table 1).

### Reliability and factor analysis

The 10 items of the CPSC scale showed good internal consistency (Cronbach´s alpha = 0.86) (Table 3). The item difficulty (1.77 [mean value]/3) was 0.59; all values were in the acceptable range from 0.2 to 0.8, indicating that there were neither ceiling nor bottom effects.

**Table 3:** Mean values and reliability analysis of the CPSC scale

**identifying item numbers and phrasings**	**Mean** ± **SD** [0–3]	**Corrected item - scale correlation**	**αif item deleted(α =.855)**	**Factor loading**
F10 In difficult situations I can observe any kind of arising emotions from a distance without getting lost in them	1.73 ± 0.82	.638	.835	.742
F12 I experience moments of inner peace and serenity even if faced with pain and disturbances or when I am dealing with difficult situations.	1.40 ± 0.84	.625	.836	.727
F3 Once I realize that my concentration has drifted I can return to consciously paying attention without difficulties	1.96 ± 0.91	.624	.836	.717
F9 I am friendly to myself when things go wrong.	1.52 ± 0.86	.583	.840	.687
F11 In difficult situations I do not let arising emotions take control over me	1.83 ± 0.85	.583	.840	.687
F6 I see my mistakes and difficulties without judging them	1.84 ± 0.82	.577	.841	.670
F8 I also accept unpleasant experiences as being important and of value.	2.13 ± 0.84	.539	.844	.641
F7 In everything I do I am paying full attention and perceive everything mindfully.	1.93 ± 0.73	.531	.845	.635
F2 With everything I do (eating, cleaning, conversations, official duties, etc.) I am always conscious of the emotions, moods and physical responses that occur	1.85 ± 0.95	.474	.850	.563
F1 I consciously perceive my current situation and can look at it (as if from the outside) without judging it as either ‘good’ or ‘bad’.	1.48 ± 0.90	.450	.852	.527

Extraction of the main components (Eigenvalue > 1); varimax rotation with Kaiser’s normalization.

Factor analysis of the 10 items revealed a Kaiser-Mayer-Olkin value of 0.893, which as a measure for the degree of common variance, indicating its suitability for statistical investigation by means of principal component factor analysis. Exploratory factor analysis pointed to one main factor (eigenvalue 4.4), which accounted for 44% of variance (Table 3).

### Correlation and regression analyses

The CPSC scores correlated (p < .0001; Pearson, 2-tailed) moderately with *Positive Life Construction* (ePLC; r=.46) and with life satisfaction (BMLSS-10; r=.38), and negatively with soldiers´ perceived stress (PSS-6; r=−.34) and self-perceived health affections (VAS; r=−.36). Moreover, we observed weak correlations with the PTSD scores (PCL-M; r=−.27) and with team satisfaction (r=.28) and self-ascribed development of strategies to cope with burdening experiences (single item: r=.26).

Because several variables which might have an impact on the CPSC scores were empirically observed, we performed multiple regression analyses to identify significant predictors. As shown in Table 4, soldiers´ PTSD symptoms (negative) and the development of effective strategies to deal with disturbing pictures and experiences (positive) were the best predictors of soldiers´ CPSC. As the regression coefficients may be compromised by collinearity, we checked the Variance Inflation Factor (VIF) as an indicator for collinearity. A VIF higher 10 is indicative for high collinearity. Results suggested that VIF was not present in the respective models. However, in several cases the VIF values ranged up to 2.7 indicating very low but tolerable collinearity in the data.

**Table 4:** Regression analyses with CPSC scores as dependent variable

**Predictors (R**^2^**=.363)**	**Beta**	**T**	**p**	Collinearity statistics *	
				**Tolerance**	**VIF**
(constant)		2.623	.010		
Perceived Stress Scale (PSS-6)	.071	.577	.565	.436	2.296
**PTSD Scores (PCL-M)**	-.255	−2.092	.039	.436	2.293
Life satisfaction (BMLSS-10)	.207	1.553	.124	.365	2.738
Positive Life Construction (ePLC)	.144	1.180	.241	.439	2.278
Self-perceived Healthy affections (VAS)	-.071	-.532	.596	.370	2.699
Psycho-emotional affections / healthy	-.037	-.396	.693	.734	1.363
**Developed effective strategies**	.174	2.068	.041	.918	1.089

* As the regression coefficients may be compromised by collinearity, we checked the Variance Inflation Factor (VIF) as an indicator for collinearity. VIF > 10 is indicative for high collinearity.

### Mean CPSC scores within the sample

**Figure 1:**
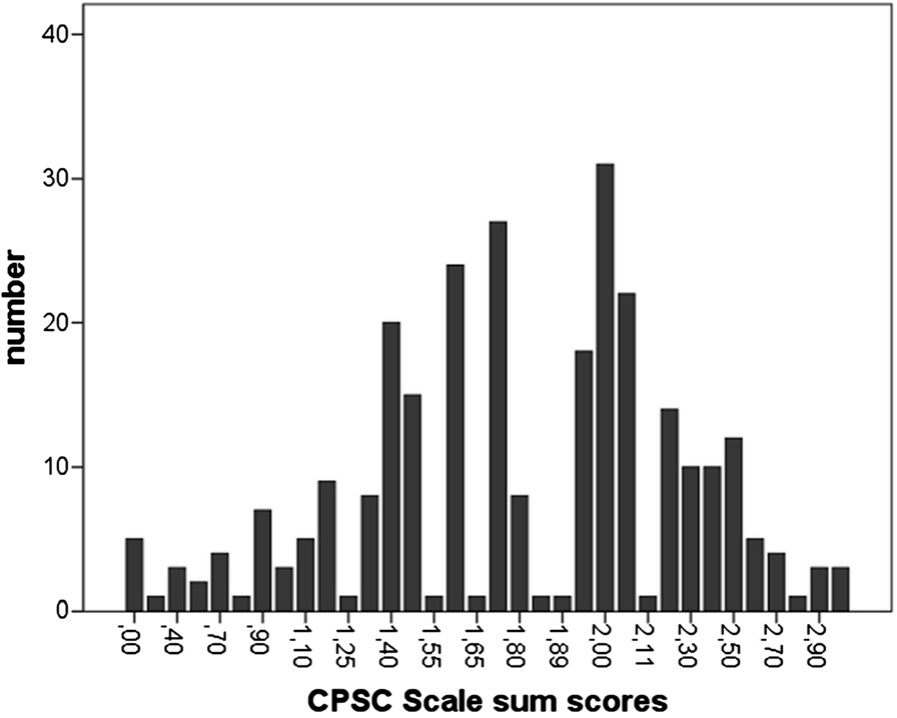
Distribution of CPSC scores within the sample [range 0 to 3].

The CPSC sum scores showed a Gaussian distribution within the sample (Figure 1). The 25% percentile was at 1.5, the 50% percentile at 1.8, and the 75% percentile at 2.1.

The mean CPSC scores (1.77 ± 0.56) did not significantly differ with respect to age (F=1.3; n.s.), family status (F=0.3; n.s.), educational level (F=0.5; n.s.) or religious orientation (F=1.8; n.s.), while they did differ between healthy individuals (1.82 ± 0.52) and patients with PTSD (1.44 ± 0.66) (F=17.3; p < 0.0001).

Only 20 individuals of the clinical sample suffered from physical trauma, and their mean CPSC score did not significantly differ from those of others (F = 0.0; n.s), while those with (self-reported) psycho-emotional affections (n=65; 1.58 ± 0.59) had significantly lower scores than the other individuals (F=9.6; p=.002). Moreover, those who stated that their affections would still exhibit impact upon their daily life had lower CPSC scores than those who were not impaired (very impaired: 1.43 ± 0.64; somewhat impaired: 1.65 ± 0.38; not at all: 1.84 ± 0.55; F=8.1; p < 0.0001).

Those soldiers who stated that they have developed effective strategies to deal with disturbing pictures and experiences had significantly higher CPSC scores than those who stated that they did not (F=4.9; p=0.001) (Table 5). The soldiers with more effective coping skills showed a trend to lower PTSD scores than those with low coping skills (F=2.2; p=0.075).

**Table 5:** Development of strategies and connection with CPSC and PTSD symptoms

**“I have developed effective strategies to deal with disturbing pictures and experiences”**	**CPSC**	**PTSD Scores (PCL-M)**
does not apply at all (18%)	1.6 ± 0.6	36.1 ± 16.6
does not truly apply (10%)	1.7 ± 0.4	35.0 ± 15.0
neither yes nor no (37%)	1.7 ± 0.5	29.5 ± 13.7
applies quite a bit (27%)	1.9 ± 0.5	30.0 ± 12.2
applies very much (9%)	2.1 ± 0.5	30.6 ± 13.9
All individuals (100%)	1.8 ± 0.54	31.4 ± 14.2
F value	4.9	2.2
p value	.001	.075

Results are mean ± standard deviation.

## Discussion

It was the intention to develop an instrument that is able to measure the ability to exhibit conscious presence as well as to maintain self regulation of individuals, who are prone to experience critical and stressful situation such as soldiers, fire fighters, emergency units etc. For that purpose we have used the Freiburg Mindfulness Inventory (FMI) as a platform for developing the *Conscious Presence and Self Control* (CPSC) scale as a proxy measure of mindfulness and situational awareness and validated the scale in a population of N = 281 soldiers. The final scale consisted of 10 items and had a good internal consistency (alpha = 0.86), and no ceiling or bottom effects could be revealed.

In contrast to the FMI with its two underlying components `acceptance´ and `presence´, the new instrument, the 10-item CPSC, exhibited a unidimensional structure. Nevertheless, the facets `acceptance´ and `presence´ can still be identified, but only as interrelated constructs. In fact, four items of the instrument refer to a conscious perception of a given situation, which is faced with full attention and concentration (items 1,2,3,7); a related item addresses the ability to experience “moments of inner peace and serenity” even in difficult situations (item 12). These five items can be related to the mindfulness sub-construct `conscious presence´. In fact, four of these items (1,2,3,7) refer to the FMI´s `presence component´ [25]. Three further items address the observing acceptance of experiences without a strong emotional involvement (“from a distance”) (items 8,10,11); two related item addresses the ability to see own mistakes and difficulties without judging them and to be “friendly” to oneself even when things go wrong (items 6,9). These five items are related to the sub-construct `self-control and acceptance´. In their original wording, these items refer to FMI´s `acceptance´ component [25]. Both underlying concepts (i.e., `conscious presence´ and `self control and acceptance´) are of importance for soldiers´ performance, particularly when they are in “peace keeping missions” facing violence, suffering and death.

To measure mindfulness in veterans, Kearny *et al.*[8] relied on the *Five Facet Mindfulness Questionnaire*[36], which uses 39 items to address non-reactivity to internal experience, observing internal experience, acting with awareness, describing internal experience, and non-judgment of experience. In Kearny´s sample, Cronbach´s alpha was .93. In contrast to the *Five Facet Mindfulness Questionnaire*, the CPSC has a good internal quality too, but is much shorter and focuses exclusively on the aspects of presence and acceptance that are related to situational awareness.

In order to assess whether or not the CPSC scores may differ in soldiers with and without psychic affections, we have enrolled `healthy´ soldiers and those with psycho-emotional affections. Of importance was the fact that affected soldiers had significantly lower CPSC scores than their putative healthy counterparts. Similarly, in case these self-reported affections still do exhibit impact upon their daily life, their CPSC scores were significantly lower as compared to those without daily life affections. In fact, the highest correlation was found between the CPSC and soldiers´ *Positive Life Construction* that refers to the ability to create and/or to maintain a stable situation in life even despite health affections, and to feel well and satisfied with the respective situation. Consequently, CPSC correlated moderately also with soldiers´ life satisfaction, and negatively with perceived stress and health affections. Thus, soldiers´ `mindfulness´, conceptualized as *Conscious Presence and Self Control*, is related to their life satisfaction and mental health. Nevertheless, individuals with such an attitude are not protected against emotions and disturbing perceptions, rather they may be able to utilize more efficient coping strategies. Indeed, soldiers who stated that they have developed effective strategies to deal with disturbing pictures and experiences had significantly higher CPSC scores than those who stated that they did not, suggesting that they don’t suppress but rather reframe their intrusions. Regression analyses indicated that the (self-perceived) development of such strategies was a significant predictor of soldiers´ CPSC, and also their PTSD symptoms. However, the causality of these associations remains unclear. One may consider the possibility that the development of effective coping strategies may results in higher CPSC and positive life construction. One may alternatively speculate that soldiers with high pre-mission CPSC may have lower mission-associated stress and cope better with PTSD symptoms than less `mindful´ individuals. So far one may suggest that high CPSC scores could indicate an ability to adapt to stressful situations, and could further be an indicator of resilience. These issues have to be addressed in future studies.

## Conclusions

The current findings can be regarded as a first step to the validation of a psychometrically sound instrument, which intends to measure *Conscious Presence and Self Control* in soldiers. Buddhist conceptualizations of `mindfulness´ are not necessarily identical with military `mind fitness´, and thus we attempted to adapt the conceptualization of `mindfulness´ to the theoretical framework of situational awareness, thereby aiming to retain the sub-constructs `acceptance´ and `presence´. Whether fostering soldiers´ mindfulness is of relevance for their performance during the missions and subsequent coping strategies at all, is not topic of this analysis. Nevertheless, our study suggested that the instrument has good quality indices and thus can be used in interventional studies. To draw valid conclusions, further analyses with a focus on soldiers with physical and mental affections are currently underway.

## Competing interests

For this study, none of the authors received any financial support. AB is employee of the Witten/Herdecke University, FZ and NK are employees of University Munich, HW is employee of the Europa University Viadrina, MT is member of the French Armed Forces. We disclose any competing interests.

## Authors’ contributions

AB initiated the project, analyzed and interpreted the data, and has written the manuscript. MT contributed to initiate the project, and contributed to interpretation of the data. HW, NK, and FZ contributed to data interpretation; NK contributed to write essential parts of the manuscript. All authors have read and approved the final manuscript.
